# Educational Concepts of Digital Competence Development for Older Adults—A Scoping Review

**DOI:** 10.3390/ijerph20136269

**Published:** 2023-06-30

**Authors:** Marielle Schirmer, Katharina Dalko, Dietrich Stoevesandt, Denny Paulicke, Patrick Jahn

**Affiliations:** 1 Faculty of Medicine, Martin-Luther-University Halle-Wittenberg, Dorothea-Erxleben-Lernzentrum-Halle (DELH), Magdeburger Straße 12, 06112 Halle (Saale), Germany; dietrich.stoevesandt@medizin.uni-halle.de; 2 Health Service Research Working Group|Acute Care, Department of Internal Medicine, Faculty of Medicine Martin-Luther-University Halle-Wittenberg, University Medicine Halle (Saale), Ernst-Grube-Str. 40, 06120 Halle (Saale), Germany; denny.paulicke@medizin.uni-halle.de (D.P.); patrick.jahn@uk-halle.de (P.J.); 3 Department of Medical Education, Akkon University of Human Sciences, Colditzstraße 34–36, 12099 Berlin, Germany

**Keywords:** digital competences, education, older adults

## Abstract

The digital transformation of healthcare and nursing is becoming increasingly important due to demographic change and the growing shortage of skilled workers. In order to ensure the participation of senior citizens in digital assistive technologies, educational concepts and support services are needed to promote digital skills in older adults. Therefore, the specific needs and prerequisites of this target group have to be taken into consideration. This paper asks how educational programs for the support of digital competences of older adults are designed and implemented. A scoping review was conducted to systematically extract existing findings from the literature. Four databases (Cinahl, PubMed, Web of Science Social Sciences Citation Index (SSCI), ERIC) were searched using an exploratory strategy to identify studies that address educational concepts promoting digital competences for older adults. A total of 47 publications were included in the qualitative analysis and show a variety of strategies to deal with the promotion of digital competences for elderly people. In conclusion, programs dealing with the promotion of digital competences for elderly people should be flexibly adapted to the target group with its specific needs and challenges such as fears, lack of previous experience, or physical limitations. For successful implementation, social support is of outstanding importance.

## 1. Introduction

### 1.1. Background

As a result of the ongoing digital transformation, digital technologies and services are increasingly determining broad areas of everyday life, including administrative processes and even the provision and implementation of healthcare [1,2]. Technological progress promises many advantages such as improved family and social connectivity, better access to information, engagement in leisure activities, as well as being able to manage health and day-to-day errands despite limited mobility [3]. Older adults, especially, are more likely to be digitally excluded than younger ones [4,5]. The technical usage behavior of older people is influenced by different variables including gender, formal education level, occupation, financial resources, milieu affiliation, generation, and images of age [6]. While an increasing digitalization of the 65+ age group can be observed, still only 37% of senior citizens in Germany—only one in three—are using the internet. However, the age group in question is not fundamentally opposed to digital technologies [5]. The ambivalent attitudes toward the topic of digitalization clearly point to the heterogeneity of people and the need to build on the general interest and create new references to knowledge and experience. Even if a growing usage behavior can be seen in the age group, the digital divide will continue to exist due to rapidly advancing technology development and the already existing variability of social inequality previously described [6].

Digital technologies can support the everyday lives of older people and contribute to a better autonomy, well-being, and quality of life as well as reduce loneliness and social isolation [2,7,8,9,10]. Still, there is a need for support programs to promote digital competences of older adults [5,11]. The domestication of technology—that means the adoption of technology in daily life—depends on individual digital competence [12]. Digitalization leads to far-reaching changes in all areas of our daily lives. Therefore, being digitally competent is essential to participate in society [8,13,14,15]. Extensive digitization of areas of life and constant changes in technical possibilities increase the pressure on all age groups to become familiar with digital innovations. The goal of the German government’s social policy is to enable people of all ages to use digital devices and services and to contribute to an active life and participation [8]. To this end, user-centered design is increasingly influencing technical developments by actively integrating end users such as senior citizens into the development processes. Thereby, the usability and applicability in the living environment of the people affected plays a central role [16,17]. In order for older people to actually benefit from the digital transformation, access to and skills to use digital technologies must be ensured. Only then can the digital transformation be a meaningful contribution to a self-determined lifestyle in old age [3,8].

Digital competence is one of the eight key competences for lifelong learning and is defined as follows: “Digital competence involves the confident, critical and responsible use of, and engagement with, digital technologies for learning, at work, and for participation in society. It includes information and data literacy, communication and collaboration, media literacy, digital content creation (including programming), safety (including digital well-being and competences related to cybersecurity), intellectual property related questions, problem solving and critical thinking” [18].

Casselden (2022) emphasizes the need for a suitable learning environment for successful digital literacy training for the elderly which addresses and reduces fears in dealing with technology and highlights its usefulness and simplicity [19]. When people reach retirement age, organized access to education ceases to exist. The continuity of education after retirement, then, depends on the intrinsic motivation of the individual and the available support [5].

In the post-professional phase of life, educational processes have the task of further developing the personality, enabling people to take on civic engagement and to be able to participate in socio-political life in a mature manner [20]. The learning processes and environment should, in the sense of geragogy, be adapted to the needs of the users and integrate existing elements of the living environment. Learning processes in old age should contribute to participation and involvement, be linked to what is known, be meaningful, promote personal initiative, be carried out in a community, and promote a sense of belonging [21]. According to the German Ageing Survey, people of retirement age in 2020/21 participated proportionately less often in courses or lectures than working people, but when they do, they do so with greater frequency [22]. In Germany, there is a large number of providers and initiatives that offer educational programs in the context of digital literacy development for seniors [5,6]. The review by Ehlers et al. (2016) on the inventory of projects, initiatives, and programs operating in Germany reveals a great wealth of ideas and creativity in their design and general recommendations for action. However, information on successes and difficulties encountered by the actors is rare. Furthermore, Ehlers et al. (2016) name a lack of systematic evaluation and internal quality control [6]. An overview of projects and initiatives operating internationally can provide a valuable addition to the existing national evidence. In the international context, there is already a wealth of evidence for the implementation of digital literacy education programs for seniors. However, a systematic overview of findings on the conceptual development of educational programs for the age group is presently not available. This scoping review, therefore, summarizes these findings, places them in the scientific context and provides a detailed and broad overview of best practices and experiences. Specifically, the review addresses the development, structure and methodological implementation of digital literacy education programs.

### 1.2. Objectives and Aim

The aim of the following literature review is, therefore, (1) to obtain an overview of international research that deal with the education of older adults in the context of digital competences and (2) to obtain criteria for the development and implementation of an educational concept from the findings.

The central research question of this review is as follows: “What findings exist for the development of an educational concept/training offer for the support of digital competences of older adults?”

Furthermore, in the analysis, attention was paid to whether statements on the participatory involvement of the participants could be found and how this was designed. While the focus was placed on design processes and criteria—i.e., setting, didactic contents, as well as the educational concept (e.g., design, theory, methodology, and format), the social support and thematic supporting factors and barriers, the research question can be further divided into the following sub-questions.

1. How are selection and development processes of didactic concepts characterized?
2. Which design criteria for educational formats (setting/materials/topics/trainer) can be identified?
3. Which criteria and recommendations for the implementation of educational programs to strengthen digital competences of older adults can be derived from the evaluation results of identified studies?

## 2. Materials and Methods

We conducted a scoping review using the methodological approach of Elm et al. This method was chosen in order to give a broad overview on existing findings and identify established principles and criteria in the international research on educational concepts in the context of digital competences for older adults [23]. It will be reported using the Preferred Reporting Items for Systematic Reviews and Meta-Analyses, an extension for Scoping Reviews (PRISMA-ScR) guidelines (Supplement Table S1). A priori, a review protocol was developed, but not published or registered.

### 2.1. Search Strategy

In the period from June to September 2022, a sensitive database search was conducted using the databases PubMed, Cinahl, Web of Science Social Sciences Citation Index (SSCI), and ERIC, which was carried out independently by two individuals (MS and KD). According to the search components—Population, Concept, and Context—the following terms searched using Boolean operators, truncations, and proximity operators:

- Population: older peoples, older adults, elderly, senior citizen
- Concept: digital literacy, digital competence, digital skills, digital inclusion
- Context: education, train, learn, support, training program, learning program, course.

The named search terms were applied in the same way in Cinahl, Web of Science, and ERIC; in PubMed only the Mesh-Terms—aged: 65+ years, 80 and over, 80+ years—were used to classify the population. All types of studies published in the period between 2012 and 2022 and available in English or German with available abstract were included. Additionally, the reference lists of the included ones were searched for further evidence (MS). All articles were merged in the web-based tool “rayyan” [24] and filtered by titles, abstracts, and full texts regarding their content fit to the research question.

### 2.2. Study Selection

The inclusion and exclusion criteria are listed in Table 1 below. Publications were included that contained a concrete project and intervention description for teaching digital skills to older adults (age 60 and older). In addition to older adults, studies that focused on the perspective of teachers were also included. Studies in an inpatient and clinical setting were excluded.

### 2.3. Data Extraction and Synthesis

The characteristics of the references were mapped in a pre-consented data form (MS) and summarized narratively. The analysis of the references took place with respect to the focus set, i.e., the references were not discussed but were structured thematically and reported in terms of evidence synthesis.

## 3. Results

### 3.1. Selection

**Figure 1:**
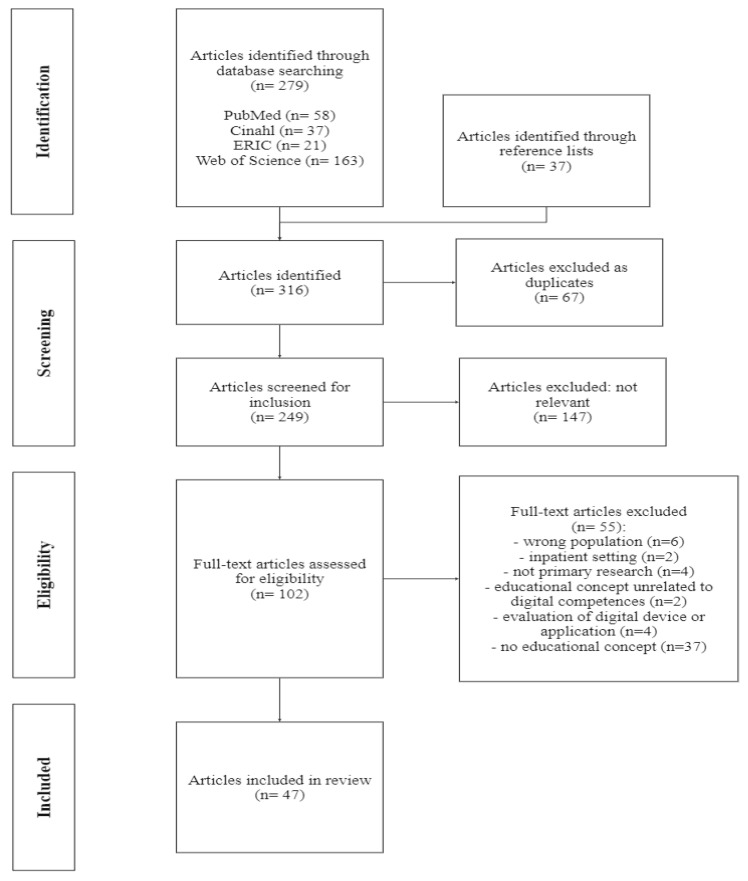
PRISMA flowchart of the study selection process.

After duplicates were removed, 249 identified abstracts according to above listed criteria of eligibility were independently reviewed (MS and KD). A full text screening of the resulting 102 publications led to 47 studies which were included in the qualitative analysis. Figure 1 shows the process of study selection in a PRISMA flowchart.

### 3.2. Characteristics of Included Studies

In the following, an excerpt of the results from the 47 included publications is presented (Table 2). The overview is intended to show which types of studies were included. Included studies (a) present results from own interventions, where self-initiated learning programs are presented and their evaluation and experiences are dealt with, (b) reports from the observation of already existing learning offers, and (c) investigations of specific learning methods, independent of a concrete learning program.

More than half of the publications (n = 27) were published in the period 2019 to 2022, showing an increase compared to the period 2012 to 2018. A total of 23 of the included publications were from Europe, 12 from the USA, 4 from Asia, 2 from Mexico, 2 from Australia, 2 from Brazil and 2 from Canada. A total of 23 of the included studies used qualitative research methods, 19 used a mixed-methods study design, only 3 used quantitative methods, and 1 used a purely theoretical approach.

One systematic review was included in the data analyses. Gates and Wilson-Menzfeld (2022) identified overlaps in the themes of negative perceptions of aging, the learning environment, and the value of technology in the studies reviewed and argue that taking these into account contributes to the improvement and more sustainable establishment of programs. Furthermore, they argue for the integration of geragogy as a theoretical basis in program development. Geragogy focuses on empowerment, autonomy, peer learning, and self-control of older people’s own knowledge. In the sense of geragogy, negatively internalized perceptions are to be questioned and dismantled. The importance of peer learning is also mentioned, but intergenerational approaches are seen as an opportunity to actively question and counter stereotypes that exist across age groups. In particular, the relevance of building self-confidence and strengthening the sense of self-efficacy of older people should be addressed from the beginning of the learning process. They also see a great opportunity in the cooperation with other organizations. Sharing common knowledge and identifying resources offers the opportunity to facilitate program development and a co-creative and participatory approach [25]. Gates and Wilson-Menzfeld (2022) focused the role of geragogy and critical geragogy in digital skills programs [25]. They explore programs in three generated themes—negative perceptions of aging, learning environment, and value of technology—but not in detail, for example, in setting, development of the programs, or teaching-methods. A detailed approach is relevant, should contribute to the derivation of conceptual criteria and support the creation of a didactic concept.

### 3.3. Synthesis of Results

The data extraction was guided by the following criteria: (1) definition of digital competences; (2) design and development process of the programs; and decisions about their design according to (3) format; (4) setting; (5) digital devices; (6) application and topics; (7) social support; (8) resources; and (9) learning environment. Finally, reference is made to (10) impact of the programs in terms of facilitating factors and barriers.

#### 3.3.1. Definition of Digital Competences

In the context of the evaluation of the available studies, a multitude of definitional approaches to digital competence could be identified. Various terms and differentiations, such as digital skills, digital literacy, information literacy, or e-health literacy, describe the skills required to use and understand digital technologies and information. Not all studies define these concepts [1,2,11,19,26,27,28,29,30,31,32,33,34]. One reason for this is the respective focus of the publication, such as meeting the digital divide, lifelong learning, or addressing specific learning approaches such as intergenerational learning or tackling digital fears. Airola et al. (2020) also refer to the EU definition and point out the lack of social and contextual references [12]. Access to digital technologies, their adoption in everyday life, and the development of competences of older people depend on their everyday life references and social patterns of meaning.

Basically, the definitions take a holistic, multidimensional view of digital literacy. Blažič and Blažič (2018 + 2020) and Grynova et al. (2020) refer to the technical, cognitive, and social-emotional dimensions of the concept of digital literacy [9,10,35]. Grynova et al. (2020) define digital literacy as “the ability of a person to perform specific activities and use digital technologies, namely: the ability to use, access, filter, evaluate, create, programme and exchange digital content; the ability to communicate, solve problems on the Internet, protect information, personal data, and use digital devices” [35] (p. 113). In the example of Blažič and Blažič (2020), the definitional approach serves as the basis for the modular development of the training concept. Thus, the technical dimension involved learning how to use the tablet, the cognitive dimension consisted of the playful acquisition of the touchscreen tablet (rules of the game, solving tasks), and the social level was taken up in the initiation of a collaborative learning environment [9].

In the definition of digital literacy, some focus can be identified—(a) a technical or application-related reference, such as the ability to use applications like ICT and to understand their function [7,36] or (b) information and media literacy, which describes finding, reflecting on, and critically appraising information from digital sources [13,37,38,39,40]. Here, the aspect of e-health literacy, which is particularly emphasized by some publications [41,42,43], i.e., the ability of a person to find, understand, and use digital health information in a reflective manner, is found again. The aspect of ensuring (c) social participation and connectedness is also taken up [12,13,15,44].

#### 3.3.2. Design and Development Process

The selection of learning content should be based on the needs of the learners, address specific everyday problems as well as life circumstances, and take social processes into account [1,3,11,14,19,27,40,45,46,47,48,49,50,51,52,53]. This can be done by involving them in the participative development process itself. To realize this, needs assessment or co-creative workshops can be identified in which the content of the curriculum is discussed and debated with the target group in advance [1,27,40,47,48,50,52,53,54]. Barrie et al. (2021) identify the direct involvement of older people as key to creating a positive learning environment and confronting ageism [13]. Older people take on an active creative role, e.g., in senior centers, actively influence existing offers and actively participate [33]. Furthermore, the previous experiences and abilities of the recipients must be taken into account in order to recognize the level of competence at which the participants are [1,26,48,54].

In the evaluation, several theories can be found that have guided the development of the learning offer—Bandura’s Social Cognitive Theory (SCT) [34,45,55], the Attentional Control Theory (ACT) [35], which addresses the reduction of anxiety in order to release cognitive resources for learning, as well as geragogical principles [14,15,36,48]. The gamification approach [9,49,56] as a mediation/learning concept can be found as well as interest-led [40,47,51] and problem-based approaches [2,35].

In three studies, the procedure in knowledge transfer is described and, thus, a possibility of transparency is created. As already mentioned in the definition section, Blažič and Blažič (2018 + 2020) derive their approach from the three dimensions of digital literacy [9]. Steelman and Wallace (2017) describe a five-stage tutoring process divided into Introduction, Triage, Planning and Preparation, Implementation, and Conclusion [55]. A working relationship is established between the participants and the tutor, and motivation to use technology and the level of digital literacy is assessed; then, a plan is developed that incorporates individual skills and resources. Problem-solving follows and ends with a summary of the outcome and the creation of the further learning plan. The Assure Teaching Model, on the other hand, consists of six phases that are structured similarly, but with the addition of the aspect of goal setting [15].

A variety of methods are used in the transfer of knowledge of digital competences or result from the experiences of the studies, which should be taken into account in the creation of an own concept. The learning pace must be appropriate so that participants have enough time to complete tasks, ask questions and design their own solutions to problems see e.g., [1,3,14,39]. Clear instructions, simple language [1,3,11,37,39], and time to review relevant aspects of learning [3,12,14,37,46,48,50,51] are recommended.

The participants support their learning process through analogue methods—notes are taken on action steps so that they can also use them at home and recall the content. The researchers pointed out that this variant is not very scalable and sustainable, as action steps can change quickly due to technological change [38,52].

Observational learning and modeling is found in 10 studies [1,19,34,37,38,45,46,48,50,55]. The instructor demonstrates behaviors and strategies, explaining their actions step by step so that the participants can understand their actions. Ma et al. (2020) and Steelman and Wallace (2017) refer to the use of different models [34,55]. The demonstration can be done by tutors of different age groups as well as by the participants themselves. In the second variant, aspects of peer learning in a group and the sharing of effective methods that have proved successful with participants [29] are taken up.

Formulating goals that are precise and moderately challenging should be done together with participants to promote their self-efficacy in the learning process [3,39,40,45,49,51,55]. To this end, Steelman and Wallace (2017) recommend targeted reassurance of reasons for a person’s interest in learning [55]. This requires special attention to the individual problems of the participants, the relevance of identifying needs and structuring learning content according to these [29,37,39,40,49,51]. Basically, the organization of content and module should be concrete enough so that a structure is apparent but also have the flexibility to allow for individual questions and room for learner participation in the learning process [14,25,27,39,50,53].

Following Bandura’s SCT, the relevance of the perceived self-efficacy of the participants is emphasized, i.e., the conviction of a person to be able to cope with future situations and to organize and carry out courses of action. The teaching of contents should be practice-oriented and the learners should be able to carry out demonstrated actions independently. It can be helpful if tutors work with learners to find solutions to challenges in the learning process and communicate missing knowledge to learners [19,45,55]. The autonomy of participants in the learning process should be supported [37]; the use of self-guided learning assignments or homework can contribute to this. This supports independent learning and experimentation outside the formal learning environment and incorporates the skills learned into daily routines [34,39,45]. The focus on hands-on learning experiences and interactive learning methods can also be found in other studies [37,41,48,50].

Six studies supported older people’s learning through the use of games [9,10,14,43,49,51,56]. Game-based learning aims to achieve learning outcomes with the help of game-based content. Individual game-based components (badges, levels, avatars) in the sense of gamification, but also full-featured games—serious games—can be used [57].

Blažič and Blažič (2018 + 2020) used a two-phase process in which participants were first allowed to play games such as puzzles and card games on a tablet and then received smartphone training [9,10]. The participants received these lectures twice a week over a period of one month. The control group only received the smartphone training without the game option. The participants who were able to use the games beforehand had fun while learning the technology, which supported the development of positive emotions. This group was able to cope with more complex tasks in the smartphone training more efficiently. The use of digital technologies was learned faster (with regard to the acquisition of skills) and more successfully. The researchers supported the learners through demonstration, explanation, or instruction but also used the aspect of collaborative learning in the group [9]. The game supports touch movements on the touchscreen as well as dexterity [14]. Games were used that the participants already knew from the analogue sphere and therefore the rules were familiar to them [9,10,56]. Playing games thus contributes to a positive initial experience for the learners and reduces fears of familiarity with the newly learned technique; the effect of positive initial experience is also emphasized in other studies [30,38].

Steelman and Wallace (2017) describe methods to reduce older people’s fears about technology [55]. For example, asking specific questions encourages a person to formulate problems in their own words. Another example is user-centered instructions, in which participants formulate their solutions in their own words.

#### 3.3.3. Format

Digital competence courses were usually scheduled as 90 to 120 minute workshops and took place one to two times a week. As a result of needs assessments and evaluations, it became apparent that a continuous offer of support is necessary in order to be able to deal with newly arising questions and problems as needs and interests evolve and technology is constantly further developed [2,11,14,19,25,26,34,41,43,46,47,50,51,53]. The courses consisted of smaller groups of five to ten people, whereby care was taken to group together participants with similar previous knowledge as far as possible [1,14,19,25,26,42,45,55]. For example, courses were offered at different levels (beginner, advanced, expert) [50], basic knowledge was built up in introductory courses to bring participants up to the same level [40], and existing knowledge was incorporated into courses [2]. In addition, participants were able to contribute their own interests and questions within the course [46,51]. Personal support by tutors was further identified as a particularly important factor in the design of the format, which motivated participants, gave them security, and compensated for possible mistakes [9,11,29,46,53].

Digitally supported formats were chosen in only a few studies. Only one study adopted the full implementation as a remote setting [12]. A blended learning approach, i.e., a combination of face-to-face sessions and additional digital learning opportunities such as learning platforms [3,14,15,19,32,50,51] and exercises, proved to be more practical than remote programs as participants might lack the skills and technical requirements to follow remote learning [50].

#### 3.3.4. Setting

Digital literacy training for older people is provided in private and/or public spaces. In some projects, the private home environment (sheltered housing) of the participants was addressed as a learning location [3,7,12,19,27,28,31,43,44,48,54,58]. In addition to outreach programs in which a contact person imparts content, e.g., [27], blended learning formats could also be found, which support a mix of learning opportunities in public spaces as well as independent learning of the age group in their own homes, e.g., with the help of e-learning programs [14,19,51]. Only one project could be identified that exclusively addressed the digital space. In addition to personal access, i.e., existing technical equipment (PC, laptop), prior knowledge was relevant as a prerequisite for participation in the program [7]. In the public space, educational offers are taken over by libraries, adult education facilities, “third age university”, and “drop-in centers” [13,30,33,41,55]. In addition, educational services are offered in elderly leisure centers or community centers for senior citizens [26,28,30,33,34,39,40,42,46,47,49,53,56,59]. Barrie et al. (2021) identify libraries as being uniquely positioned to respond to the growing need for digital literacy education for older people [13].

#### 3.3.5. Digital Devices

Roughly half of the studies provide information on the selection of devices and applications addressed in the educational concept, according to which a variety of different hardware and software was included. Mobile digital devices such as tablets and smartphones were the most frequently introduced as subjects [3,14,34,38,39,40,46,47,48,53,54] while a majority of studies incorporated more than one device. Others simply stated that programs were focusing on information and communication technology (ICT) [11,28,29,30,31,35,54]. Computers are rather insignificant and were addressed by only four studies [26,40,41,50].

Another central approach is the bring-your-own-device principle, according to which participants can bring along their own technical devices and raise their specific questions and problems [35,42,47]. This approach is strongly linked to methodological considerations on interest-driven educational formats. The studies by Xie et al. (2012) and Wang et al. (2015), on the other hand, were particularly designed to enhance skills related to e-health services and devices. The former focused on specific government services providing general health information, while Wang et al. conceptualized a program introducing digital devices such as blood pressure monitors or pedometers to older adults.

#### 3.3.6. Applications and Topics

Topics and applications can be divided into the main categories of basic skills, communication, social media, e-health and health information, browsing and cybersecurity, e-entertainment, online banking, and shopping (see Table 3). Basic skills include the general set up of devices and main settings but also photo and video functions and Microsoft Office tools [14,19,28,32,34,39,40,46,50,53,56]. The categories of communication and social media both relate to social interactions online. While communication refers to content such as email services, text messaging (WhatsApp), or video conferencing [3,14,19,28,32,34,40,46,50,54], social media deals with all the functions of various social networks, with a focus on Facebook [28,32,39,40,47,50,53]. Programs incorporating e-health into their curriculum cover diverse aspects from searching for health-related information and digital services such as managing appointments to digital devices from the field of healthcare [3,14,28,34,41,43,44,53,54]. The search for information on the internet [14,19,28,32,50], on the other hand, is strongly connected to the topic of cybersecurity and information literacy. Some of the main questions dealt with the identification of scams, understanding the principles of hacking, or dealing with fake news in the sense of what information can be trusted [19,28,40,50]. Lastly, leisure-related activities such as online shopping [14,19,32] and digital entertainment [14,32,34,39,53] have been topics in a few curricula.

Gamification approaches use games for the purpose of teaching certain skills, such as touch commands or typing. The content of the game application itself is of secondary importance, but is usually based on the previously identified interests of the participants [9,10,49,51,56].

On the other hand, 15 publications do not specify curricula and content design. This results, among other things, from the chosen concept, since mentoring and drop-in (bring-your-own-device) formats do not specify particular devices and applications. Only Flynn et al. and Jones et al. provide an insight into topics that participants referred to in a drop-in format. These include communication and social media skills as well as general settings and functions of different devices (computer, phone, tablet) and applications such as navigation tools, payment options, and the search for medical information [31,32].

#### 3.3.7. Social Support

Social support is one of the promoting factors for providing digital competence for older adults that were mentioned the most [2,3,11,12,13,19,45,50,52]. Social support is understood as a “qualitative property of social relationships” and can be conveyed in different forms emotionally, instrumentally, or informationally [60]. Social support enables positively perceived social contacts and a sense of social support and belonging. Support services thus contribute to a person’s orientation, enable (self-) affirmation, and strengthen resilience and control [60].

Digital competences are predominantly acquired informally. Primarily, the support of older people derives from their social network of families, friends, and acquaintances, but formal educational references are also used more [4]. Depending on the initiator and the institution organizing the educational offer, people of different age groups and professions take over the education. For example, support is provided by students or academic staff [3,9,10,15,35,38,41,42,43,45,47,48,50,53,54,55], library staff [13,19], elderly-leisure-center staff [26,37], family members [12,14,27,30], or peers [9,10,14,26,28,30,32,46,47,49,52,56,61]. Five studies indicated that the supporters were volunteers [12,28,32,47,50,52]. For the others, it was not specified whether they were full-time or voluntary. Furthermore, digital avatars [26] were named as a source of help. Peer-support was used in 12 of the included publications. Peer support is also described as important in collaborative learning among participants [14,32,46,56,61]. Also addressed has been the importance of intergenerational learning relationships [27,31,42].

The personal qualities of the instructor, such as patience, calmness, attentiveness, composure, understanding, and friendliness, are described [3,19,28,33,37]. Empathy and being sensitive to participants’ individual needs and wishes are also named [14,61]. Equally relevant is the availability of help and assistance at eye-level in solving challenges [14,56] and supports trust in the trainee–trainer relationship [3,33]. A person acting continuously can contribute to building trust [3,33,42]. The immediate availability of help can be ensured in group learning through the support of two tutors [48,53]. The role of the trainer is that of an animator and motivator who promotes independent learning through praise and encouragement, provides suggestions and guidance when problems arise, and answers questions [14,53]. Damodaran and Sandhu (2016) refer to the need to provide neutral information on products and applications [2].

Statements on the preparation of trainers for their task are only taken up in a few of the included studies. Written instructions such as manuals were used for preparation [28,46,48], and training sessions [28,32,35,36,42,43,48,53] or video tutorials were offered [42]. The duration and intensity of the training varied from an average of 3.5 h mentoring per student [42], 8 hours for 4 weeks [43], or 12 h [53]. Geragogical principles [14,36], educational gerontology [53], or problem-based and practically oriented learning approaches [35] were also taken up in the preparation of the coaches. Tomczyk et al. (2020) point out that, in addition to content-related aspects (data on software and applications), methods (learning scenarios) and psychological and therapeutic aspects—dealing with declining motivation and learning blocks—should also be addressed [36]. The initiation of learning communities among tutors can also contribute to support in the network, trigger reflection processes in their own actions, and serve to exchange helpful teaching strategies [32,36,45].

#### 3.3.8. Resources

Generally, devices needed for educational measure are provided by the organizing party in the form of loaned devices (tablets, etc.) [19,38,43,47,51,53,54,59] or access to a computer room [15,40,41,50,52]. The only exceptions here are those formats that include a mentoring or drop-in offering where participants bring their own devices (see paragraph on devices).

In addition, guidance and instructions for devices and applications as well as tasks are provided by means of handouts and manuals [1,3,15,25,39,40,41,45,46,50,51,53,54]. Thereby, a focus lies on tailoring resources to specific needs of the target group. For example, instructions are provided in a simplified step-by-step format—without complicated tech jargon—and preferably in printed form [39,46].

Furthermore, a small number of programs implement e-learning platforms and resources either as a supplement to a blended learning approach or as an independent course [14,15,58].

#### 3.3.9. Learning Environment

The establishment of a welcoming learning environment has been discussed and described in numerous studies in a differentiated manner. Initial evaluative results have shown that a motivating, familiar atmosphere can contribute to learning success and can counteract feelings of anxiety in the context of educational concepts for older people [2,19,29,45,46,52]. A suitable setting and an appropriate form of communication are both necessary to create such an atmosphere. At best, the educational program should be characterized by a supportive environment, without any pressure to perform [2,13]. An informal, familiar atmosphere in the course can contribute to the fact that problems and questions can be addressed without shyness. In this context, the development of a learning community and the promotion of exchange between the participants is also emphasized (peer-to-peer learning) [1,9,10,13,19,29,39,46,47,49,50,52].

Furthermore, it is essential to tailor the program to the target group. Ferreira et al. (2016) emphasize that “older people require different digital inclusion strategies than younger users” [30]. For example, the venue should be easily accessible and sensory deficits and other age-related limitations such as forgetfulness or slower learning pace should be taken into account when designing materials and tasks [29,30,37,50]. This is closely connected to the aspect of communication. Therefore, several studies elaborate on the need to communicate educational content with the target group of the elderly and their specific needs—including age-related deficits [37,50]—and backgrounds in mind [1,15,37,39,53,61]. Schirmer et al. (2022) suggest, for example, the use of analogies and terms appropriate to the target group [61]. Non-judgmental and inclusive language can also address fears and reservations [39].

Security of personal data has also been identified as an important criterion [49], as there is increased uncertainty in this regard, particularly among older people [1,2,54].

#### 3.3.10. Impact of the Programs—Facilitating Factors and Identified Barriers

**Figure 2:**
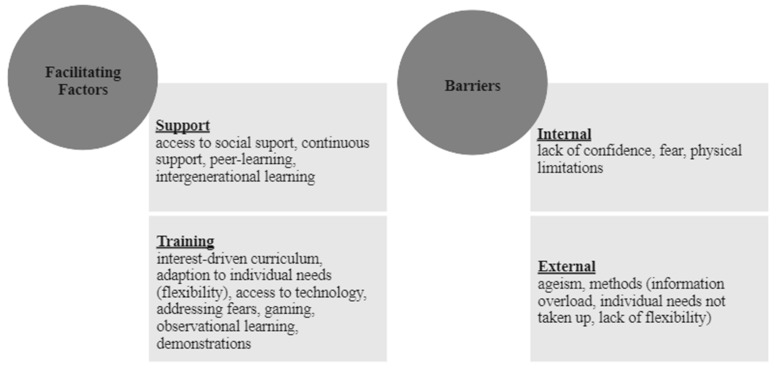
Facilitating factors and barriers.

In the following, recommendations from the included publications are compiled, which support or hinder the learning of older people in dealing with digital devices as well as the use of educational offers. Figure 2 shows a comparison of facilitating factors as well as barriers. The “Model for Promotion of ICT for Older Adults” by Barrie et al. (2021) was used as a guide. Among the promoting factors, social support in particular can be highlighted as an essential success factor of educational programs [13]. Furthermore, the recommendations refer to framework conditions and training methods. Therefore, the facilitating factors were divided into the categories of support and training. Barriers are divided into those that arise from within the individual (internal) and those that arise from the external (social context) or from the training offered.

In promoting digital literacy in the age group, access to social support and connectedness is a key success factor [2,3,12,13,45,46,47,50,52] as well as ensuring the continuity of support provision [2,19,25,44,45]. In this context, social support within the age group itself [2,28,34,47,48] as well as intergenerational approaches [25,27,31,42,54] prove to be beneficial.

Within the educational program (training), the use of an interest-led curriculum is recommended as well as the flexible integration and adaptation of the curriculum with regard to individual needs, not only at the beginning but also during the offer [2,3,29,30,40,47,48,51,52,53,54]. The provision of digital devices can also be beneficial [19,36,48] and contribute to overcoming financial access barriers. Supportive learning methods recommended are addressing participants’ fears [13,39,50], gaming [9,10,51], observational learning, and demonstration [1,34,37,38,45,48,50,55].

Internal barriers to the adoption of digital devices include lack of confidence [13,51], fear [13,36,45,47,55], and physical limitations [12,36,42,48,59]. Barrie et al. (2021) add reluctance and lack of affinity as internal barriers [13]. Furthermore, they state that internal as well as external ageism leads to impairments in people’s self-perception, self-confidence, and willingness to learn and can thus have an impact on their digital competence and social exclusion. Gender roles are also named as an external barrier [13]. Barriers in educational programs are not only ageism [1,3,12,13,25,33], but also the lack of consideration of individual needs [33,48,54], a lack of flexibility (rigid educational formats) [11,33,47], and information overload [25,61].

## 4. Discussion

This scoping review shows the importance and variety of strategies to deal with the promotion of digital competences for elderly people. The programs should be flexibly adapted to the target group with its specific needs—as shown in examples by Ferreira et al. (2016), Seo et al. (2019), and Damodaran et al. (2013) [29,30,40]—and challenges such as fears, lack of previous experience, or physical limitations, as pointed out by Atkinson et al. (2016), Steelman and Wallace (2017), and McGinty 2020 [45,48,55]. Some studies, therefore, applied co-creative or participatory development approaches to tailor these programs to the needs of the target groups and lower barriers [11,13,27,29,47,49].

As part of the scoping review presented here, studies relating to numerous diverse educational approaches and methods were included. On the one hand, this results from the explorative international and broadly chosen search strategy; on the other hand, it shows the lack of establishment of approved concepts to date. The included studies are primarily pilot projects that represent individual examples, but do not point to established best practices; see, e.g., [3]. Continuous support programs—frequently recommended in the literature reviewed in order to address ongoing and developing support needs [2,19,25]—are still either not in place or no scientific findings are available on the evaluation of such projects. Moreover, evaluation results available so far are hardly comparable due to the diversity of selected assessment instruments and methods.

A recurring requirement identified in the studies is the inclusion of specific needs of the target group itself. In addition, the preferences and interests of the participants in the programs are also addressed. Yet, only a small fraction of included studies applied participatory approaches [11,13,27,29,47,49]. Similarly, the involvement of those affected remains superficial and is usually limited to joint decision-making on individual content-related aspects. However, the general conditions, conceptual approaches, and methods are specified by the initiators. In this context, participatory approaches such as co-creation offer the potential to adequately incorporate the above-mentioned needs and requirements by actively including seniors and other stakeholders involved in educational processes in the collaborative development of new concepts [25]. In the health and nursing sciences, for example, corresponding formats are increasingly finding their way into practice in order to develop user-friendly and accepted assistive technologies. As part of this process, affected individuals themselves play a decisive role in the development of new formats and innovations [62,63].

The claim to take into account the extremely heterogeneous and individual requirements of the target group within the framework of educational programs, such as previous experience, fears, individual needs in terms of content and methods, etc., therefore contrasts with the implementation of programs in practice. Flauzino et al. (2020), for example, recommend “the combination of several teaching approaches enabling the elderly’s adoption and use of technologies, such as observational learning, collaborative learning, providing step-by-step explanations and allowing learning by trial and error […], since it [this study] has evidenced a necessary alignment between techniques and methods to be adopted by the instructors with the older students’ specific demands” [37] (p. 11).

A European case study by Muñoz-Hernández et al. (2021) also worth mentioning shows how this requirement can be addressed by using digital solutions. The study explored the effectiveness of the TechPeopleCare (TPC) methodology, a combination of the two-screen approach, instructional videos, and software environment [64]. The use of digital applications can support the learning of the target group because the users can control the learning process more individually in the learning pace and repetitions. However, an essential requirement is accessibility and usability. The use of software must be barrier-free and possible without outside assistance. Digital applications enable users to learn independently of time and space. Usually, support programs and courses aiming toward the promotion of digital competences of older adults included in this review were implemented in the context of public institutions that are not equipped for this purpose (community centers, …); see, e.g., [26,33,49,59]. Further, programs are carried out by unqualified or even volunteer personnel; see, e.g., [33]. Training courses offered in some cases for tutors and supervisors, which last only a few weeks, are hardly suitable for resolving this deficit.

In line with that, existing programs cannot be considered as inclusive. People without access to community centers who live in rural areas or experience financial barriers are excluded. Those affected are required to be active members of their communities, to network, and to seek out specific services. Cheng et al. (2022) describe this problem.

However, an entry requirement is often suggested by community-based training. For the effectiveness of such programs, instructors often admit older adults who can better engage based on their socioeconomic background, thereby excluding older adults for whom the training may be more necessary [27] (p. 3).

As already mentioned in the introduction, there is a lack of organized access to education for people of retirement age [5]. The design of inclusive framework conditions is the responsibility of the creators and providers of educational services. Especially in a time when digital competences are a key competence for participation in social life, the consideration of inclusive access requirements should be obligatory.

### Limitations

Although the scoping review was supported by steps including refinement of the protocol through team discussion, blinded searching, and selection of articles by two researchers, several limitations have to be mentioned. The present study did include gray literature where it was perceived as specific enough to fit to the research question. This was especially the case with experience reports to enhance the overall impression of the studies reviewed. Thus, it is possible that studies with a focus on human and technical interaction and digital competence have been overlooked. The search protocol was based on a combination of keywords that may not capture all relevant studies, but this allowed the focus to be maintained. Broadening the context could contribute to more hits related to digital literacy development in older people.

While the inclusion criteria of this review designate older, non-working adults aged 60 years and older, the population of some of the studies analyzed involved individuals aged 50 years and older. In the included publications, age groups such as in the example of Betts et al. (2019) from 54 to 85 years of age can be found; the program is thus directed at the target group we are aiming at and was, therefore, included in the analysis, but also involved younger people. Further, information on the occupational situation of the study population was not provided in every case.

Important to note is that study designs implemented by the included studies were very heterogeneous. Further, mainly qualitative studies with partly small numbers of participants were analyzed in this review. It has to be pointed out that one author (Damodaran) published three studies based on the same research project but with a different scope. However, the scoping review method was deliberately chosen in order to be able to explore an overview of the topic, whereas a critical evaluation of the studies was not intended. Furthermore, no long-term studies could be integrated, only cross-sectional surveys.

As mentioned in the introduction, there are many different initiatives, projects, and institutions in Germany alone that deal with the topic of digital literacy promotion in the age group; however, no scientific monitoring is carried out [6].

The theoretical and practical implications derived from this review need to be increasingly tested and validated using empirical research methods (e.g., MRC framework). Nevertheless, the strength of this narrow context is that it offers insight into certain areas and their potential.

## 5. Conclusions

This review provides an overview of various teaching formats in the field of digital competence education for older adults. Thereby, social support—especially learning from and with others—is one of the key criteria. Continuous, flexible support programs adapted to the target group are required in order to meet the ongoing and constantly changing needs. Moreover, it is important to take into account not only needs and interests of senior citizens during their learning process but also their specific challenges such as fears, lack of previous experience, or physical limitations. Awareness of ageist stereotypes and prejudices is an additional criteria and should play a central role in the design of appropriate programs in order to avoid developing them without considering the complex perspective of those affected. Furthermore, it is the task of scientific research to develop empirical, reliable findings for the sustainable, long-term implementation of support programs for older adults in the years ahead.

## Figures and Tables

**Table 1:** Criteria of inclusion and exclusion.

Inclusion Criteria	Exclusion Criteria
Studies including older adults (60+) with or without need for care as the population Studies including teachers of programs focusing on digital competences Studies dealing with educational programs to increase digital literacy among older adults	Clinical setting Population aged under 60 Population consisting of older adults who are still employed absence of reference to education or support program

**Table 2:** Overview of types of included studies *.

Author (Year)	Country	Objective	Study Design	Population	Sample Size
(a) Evaluation of original educational programs					
Arthanat et al. (2019) [3]	USA	Multi-stakeholder perspectives to identify and conceptualize barriers/strategies for effective implementation of information communication technology (ICT) training for older adults	Qualitative	Older adult ICT trainees, care providers, and ICT trainers	n = 61
(b) Findings from existing learning programs					
Barrie et al. (2021) [13]	Canada	Exploration of experiences of older adults attending digital literacy training sessions offered by the public library system	Qualitative	Older adults (age 60+)	n = 12
(c) Studies in regard to specific learning methods					
Blažič and Blažič (2020) [9]	UK, Austria, Slovenia, Macedonia	Older adults participated in a two-phase process: playing interactive games on a touchscreen tablet and learning how to use a smartphone to access digital services to support digital skills	Mixed-methods	Older adults (age 57+)	n = 146

* for the complete overview of included studies view the Supplementary Material Table S1.

**Table 3:** Overview of topics and applications.

Title 1	Title 2
Basic skills—general functions and features, device set up	[14,19,28,32,34,39,40,46,50,53,56]
Communication	[3,14,19,28,32,34,40,46,50]
Social media	[28,32,39,40,47,50,53]
e-Health, health information and services	[3,14,28,34,41,43,44,53,54]
Internet browsing	[14,19,28,32,50]
e-Entertainment	[14,32,34,39,53]
Online banking and shopping	[14,19,32]
Cybersecurity	[19,28,40,50]

## Data Availability

Not applicable.

## Supplementary Materials

The following supporting information can be downloaded at: https://www.mdpi.com/article/10.3390/ijerph20136269/s1, Supplement Table S1. PRISMA Guidelines. Supplement Table S2. Full list of publications included in scoping review.
