# Supplementary material for: Educational Concepts of Digital Competence Development for Older Adults—A Scoping Review
Source: Int J Environ Res Public Health. 2023 Jun 30;20(13):6269. doi: 10.3390/ijerph20136269 (PMC10341140; doi:10.3390/ijerph20136269)
Supplement: Supplementary file 1 [file ijerph-20-06269-s001.zip › Supplement Table S2. Full list of publications included in scoping review.pdf]

Table S2. Full list of publications included in scoping review.

Author (Year)	Country	Objective	Study Design	Population	Sample Size
Airola et al. (2020)	Finnland	Evaluating the use of phone and video conferencing service aimed at promoting the social connectedness and well-being of older people	Qualitative	service coordinator, volunteers, and service users (age 88-89 years)	n = 5
Arthanat et al. (2014)	USA	Effectiveness of a home-based information communication technology (ICT) training program for older adults	Qualitative	older adults (age ≥ 60 years)	n = 13
Arthanat et al. (2019)	USA	Multistakeholder perspectives to identify and conceptualize barriers/ strategies for effective implementation of information communication technology (ICT) training for older adults	Qualitative	older-adult ICT trainees, care providers and ICT trainers	n = 61
Atkinson et al. (2016)	USA	Experiences of learners and student tutors who participate in digital literacy sessions for seniors	Qualitative	Tutors and trainees	n = 6
Barrie et al. (2021)	Canada	Exploration of experiences of older adults attending digital literacy training sessions offered by the public library system	Qualitative	older adults (age ≥ 60 years)	n = 12
Beh et al. (2018)	Australia	Evaluation of an interest-based curriculum for digital competences of older adults in order to foster their digital inclusion	Mixed-Methods	older adults (age ≥ 65 years), staff members, teachers	n = 131
Betts et al. (2019)	UK	Defenition of digital technology and experiences of digital inclusion sessions	Qualitative	older adults (age 54-85 years)	n = 17
Bevilacqua et al. (2021)	Italy	Innovative eHealth training to improve digital literacy and a new learning culture in later-life learning	Mixed-Methods	older adults (age ≥ 50 years)	n = 58
Blažič & Blažič (2018)	UK, Austria, Slovenia, Macedonia	Evaluation of game-based learning approach to foster digital skills of older adults	Mixed-Methods	older adults (age 57-87 years)	n = 146
Blažič & Blažič (2020)	UK, Austria, Slovenia, Macedonia	Two-phase process of mobile device training in order to support digital skills of older adults	Mixed-Methods	older adults (age 57-85 years)	n = 146

Carvalho et al. (2019)	Portugal, Spain, Austria, Slovakia, Czech Republic, Italy, Romania	Evaluation of digital skills course to measure outcome and level of satisfaction	Mixed-Methods	older adults (age ≥ 50 years), trainers of digital skills class	n = 311
Casselden, B. (2022)	UK	Evaluation of the Housing Plus Pilot providing remote digital skills training and support	Qualitative	older adults (age ≥ 55 years) living in sheltered housing	n = 15
Castilla et al. (2018)	Spain	Examining a social network consisting of multiple applications with linear navigation as a digital literacy method for the elderly in rural areas	Mixed-Methods	older adults (age 60-76 years)	n = 46
Cheng et al. (2022)	China	Evaluation of the implementation of a family intergenerational learning (FIL) project	Qualitative	grandparents and grandchildren in rural areas	n = 20
Chiu et al. (2016)	Taiwan	Evaluation of an eight-week touchscreen mobile device training for older adults in a low Internet usage area	Mixed-Methods	older adults (age ≥ 50 years)	n = 20
Damodaran et al. (2013)	UK	Investigating older people's ICT learning and support needs	Mixed-Methods	older adults (age ≥ 50 years)	n = 750
Damodaran et al. (2014)	UK	Examination of older people's use of information and communication technologies (ICTs) and identifikation of the factors which can prevent or promote their sustained use	Mixed-Methods	older ICT users (age ≥ 50 years)	n= 323
Damodaran & Sandhu (2016)	UK	Exmination of the role of formal and informal social support in reducing digital inequalities by enabling the digital participation of older people	Mixed-Methods	representatives from interest groups, government officials, academics, practitioners	n > 1000
Davis et al. (2018)	Australia	Co-Design and evaluation of a skills developing model (interests-participation model) and pilot workshops	Qualitative	older adults (age ≥ 60 years)	n=19

Ferreira et al. (2016)	Brazil	Evaluation of computer classes using key informants, interactive observation techniques and drawing on computer-assisted analysis	Qualitative	older adults (age ≥ 60 years)	n = 78
Flauzino et al. (2020)	Brazil	Verification of teaching-learning process in digital literacy programmes for older adults, based on students' perceptions	Qualitative	older students (age ≥ 60 years)	n = 278
Flynn (2022)	Ireland	Exploration of perspective of young adults as parties to an intergenerational learning exchange within a family environment to support the digital skills of their older adult family members	Qualitative	university students (age 18-25 years)	n = 248
Gates & Wilson-Menzfeld (2022)	UK	Exploring the implementation and delivery of digital skills programs for middle and older age adults; and understand the presence of adult learning theory in their delivery	Systematic Review	n.a.	n.a.
Grynova et al. (2020)	Ukraine/Poland	Substantiate educational strategies of collaboration, research and mentoring skills development in preservice teachers facilitating older adults' learning of ICT with the use of problem-based practice-oriented adult learning activities	Mixed-Methods	student mentors	n = 54
Haan et al. (2021)	Netherlands	Evaluation of the implementation of an educational approach based on peer learning in a living lab setting	Qualitative	older adults (age ≥ 65 years)	n = 7
Jones et al. (2015)	UK	Evaluation of volunteer support in helping older people to go online	Quantitative	older adults (age ≥ 65 years)	n = 144
Kim et al. (2022)	USA	Examination of how older adults who had never used a tablet computer learn to use it, what they want to use it for, and what barriers they experience as they continue to use it during social isolation caused by the COVID-19 pandemic	Qualitative	older adults (age ≥ 65 years)	n = 8

Lee et al. (2019)	USA	Investigating the effectiveness of Intergenerational Mentor-Up (IMU) on levels of ehealth literacy, technophobia and social isolation of older adults	Mixed-Methods	older adults (age ≥ 65 years)	n = 55
Lenstra (2017)	USA	Examining the influence of a community-based information infrastructure of older adult digital literacy	Qualitative	older adults, library staff	n = 216
Ma et al. (2020)	China	Investigating the effectiveness of observational training through behavior modeling in enhancing technology acceptance in older adults	Mixed-Methods	older adults (age ≥ 60 years)	n = 59
Martínez-Alcalá et al. (2018)	Mexico	Evaluation of a blended workshop based on a Learning Management System (LMS) as a supporting tool for older adults' digital literacy	Quantitative	older adults (age ≥ 60 years)	n = 98
Martínez-Alcalá et al. (2021)	Mexico	Exploring the learning process of older adults aquiring ICT skills	Mixed-Methods	older adults "aged around 60"	n = 251
McGinty (2020)	USA	Developing a training program for Digital Literacy Coaches for older Adults	Qualitative	instructional design graduate students	n= 4
Pang et al. (2021)	Canada	Exploring older adults' perceptions of adopting and learning new technologies within the lens of health management support, testing prototype Design of Help Kiosk 2.0	Mixed-Methods	older adults (age ≥ 65 years)	n = 42
Rasi et al. (2021)	Sweden, Finland	Investigating learning strategies and support needs of older adults acquiring skills in regard to health care technology	Qualitative	older adults (age ≥ 60 years)	n = 19
Santos et al. (2013)	Spain	Co-design and implementation of an m-learning activity based on dialogic literary gathering offered by adult school	Mixed-Methods	older adults (on average 65 years old)	n = 20
Sayago et al. (2012)	Scotland/Spain	Examining strategies used by older adults to gain ICT skills, age related difficulties	Mixed-Methods	older adults (aged ≥ 55 years)	n = 420
Schirmer et al. (2022)	Belgium	Investigating experiences of teachers of older adults in regard to teaching strategies as part of older adult digital literacy classes	Qualitative	teachers of older adult digital literacy courses	n = 26

Seo et al. (2019)	USA	Needs assessment of older adults in order to conceptualize a digital literacy program; evaluation of the program	Qualitative	low-income African-American older adults (aged ≥ 55 years)	n = 47
Springett et al. (2022)	Slovenia, Noth Macedonia, UK	Introducing three studies using gamification methods to enhance digital skills of older adults	Qualitative	older adults (age ≥ 60 years)	n = 88
Steelman & Wallace (2017)	USA	Description of social-cognitive approach to training digital literacy skills	Qualitative	patrons and tutors	not specified
Tirado-Morueta et al. (2021)	Spain	Evaluation of usefulness of institutional support for digital literacy for older adults	Quantitative	retired adults (age ≥ 55 years)	n = 560
Tomczyk et al. (2020)	Poland	Perspectives of digital literacy educators of older adults on the digital divide	Qualitative	specialists, educators of older adults	n = 8
Wang et al. (2015)	Taiwan	Introduction of Health Education Learning Program with Science for seniors (HELPS-seniors) encouraging medical staff to use new self-management tools for technophobic elderly people	Mixed-Methods	older adults (age ≥ 65 years)	n = 48
Woodward et al. (2013)	USA	Evaluation of a peer tutor model to teach older adults how to use information and communication technologies (ICTs)	Qualitative	Older adults (age 61-85 years)	n = 19
Yoo (2020)	USA	Introduction of a six week course to enhance senior digital literacy with a special focus on smart mobile devices	n.a.	older adults (age ≥ 50 years)	n.a.
Xie (2012)	USA	Evaluation of a e-health literacy intervention for older adults	Mixed-Methods	older adults (age ≥ 60 years)	n = 218
